# Pattern of fractures across pediatric age groups: analysis of individual and lifestyle factors

**DOI:** 10.1186/1471-2458-10-656

**Published:** 2010-10-30

**Authors:** Giuliana Valerio, Francesca Gallè, Caterina Mancusi, Valeria Di Onofrio, Marianna Colapietro, Pasquale Guida, Giorgio Liguori

**Affiliations:** 1 School of Movement Sciences (DiSiST), Parthenope University, via Medina 40, 80133, Naples, Italy; 2 Department of Orthopaedics and Traumatology, Santobono-Pausilipon Children Hospital, via Mario Fiore 4, 80129 Naples, Italy

## Abstract

**Background:**

Knowledge of the epidemiology of children's fractures is essential to develop preventive strategies. The aim of this study was to analyze the individual/lifestyle determinants of fractures across pediatric age groups.

**Methods:**

A cross-sectional study was performed in the first six months of 2008 through questionnaire on a sample of children from an outpatient clinic for pediatric fractures. Differences in gender, anatomic site, circumstances and location of fracture occurrence, behavioural lifestyle, and calcium intake were investigated among three different age classes (pre-school children, school children, and adolescents).

**Results:**

The sample consisted of 382 subjects (2-14 years of age) sustaining a fracture after low or moderate trauma. Males were at a higher risk of fractures than females; greater than two-thirds of injuries occurred after low-energy trauma and the upper limb was more frequently involved. With increasing age, the male/female ratio and time spent in sports participation increased (p < 0.001), while calcium intake and time spent in sedentary behaviors decreased (p < 0.001 and < 0.003, respectively). Gender discordance existed in pre-school children with respect to the anatomic location, and in school children and adolescents with respect to the dynamics. In the adolescent group, males were more physically active and also more sedentary than females. Fractures most frequently occurred in homes (41.6%), followed by playgrounds and footpaths (26.2%), sports facilities (18.3%), and educational facilities (13.9%), with gender differences existing only in adolescence. Twenty-three percent of the subjects sustained one or more fractures in the past. The percentage of recurrent fractures increased with age (p = 0.001), with a similar trend in both genders.

**Conclusions:**

Gender differences were shown in the prevalence of injuries, characteristics, and circumstances across ages. These differences may be explained by the related changes in behaviors, together with attending different places. Individual and lifestyle factors can in part explain the variability in the occurrence of fractures and can also address targeted preventive strategies.

## Background

Fractures are extremely common in the pediatric age group, representing a major public health problem. The lifetime risk of sustaining a fracture in childhood is approximately 42%-64% in boys and 27%-40% in girls, with remarkable variation in the estimates worldwide [1-4]. While fractures more often occur in males, girls usually sustain fractures at a younger age compared to boys [2-7]. Even though several genetic, endocrine, or systemic illnesses that affect bone metabolism may cause fractures, the majority of children with fractures are otherwise healthy. Several factors have been analyzed for their role in determining fracture risk. Bone mass and bone mineral density, low calcium intake, high body mass index (BMI), inactivity, behavioral difficulties, consumption of carbonated beverages, use of drugs (corticosteroids) have been variably associated with this kind of injury in children [7-11]. It has also been demonstrated that a first fracture at a young age is associated with an increasing risk of sustaining subsequent fractures [12,13].

Studying the epidemiology of children's fractures is essential in developing preventive strategies. The importance of analyzing the etiology of injuries, and the circumstances and settings in which they occur in the various stages of development is to identify risky behaviors or an unsafe environment which can be corrected by specific preventive measures appropriate for age.

The purpose of this cross-sectional study was to analyze the individual and lifestyle determinants of fractures in a series of outpatient children and adolescents. Comparison of gender, anatomic site, circumstances and location of fracture occurrence, behavioural life style, and calcium intake were analysed among three different age classes (pre-school children, school children, and adolescents).

## Methods

This was a cross-sectional study conducted in the outpatient clinic of the Department of Orthopedics and Traumatology of Santobono-Pausilipon Children Hospital in Naples (southern Italy). This hospital is the largest children's hospital in the Campania region, providing inpatient and outpatient services in emergency and trauma medicine in children < 14 years of age within the metropolitan area of Naples. The study commenced on 1 January 2008 and was terminated on 30 June 2008. In 2008, the metropolitan area included 4,434,000 residents, of whom 17.3% (767,082 subjects) were < 14 years of age. The investigation was approved by the Ethical Committee of the Santobono-Pausilipon Hospital and written informed consent was obtained from all participants and/or their parents or legal guardians in accordance with the revised version of the Helsinki Declaration regarding research involving human subjects.

The inclusion and exclusion criteria are reported in table 1. Children < 2 years of age were not included because the analysis of lifestyle behaviors is hardly applicable at this very young age. The population was divided into 3 age ranges (pre-school children [2-5.9 years], school children [6.0-10.9 years], and adolescents [11-14 years]). Height and weight were measured and the BMI was calculated according to the following formula: (weight [kg]/height [m^2^]). Since BMI is age- and gender-related, this parameter was transformed into a standard deviation score (SDS), based upon the established Center for Disease Control normative curves [14]. Fractures were confirmed radiographically at the time of injury. Using information about each event documented in the medical record, fractures were classified based on their anatomic site, the location of the injury occurrence (home, playground, footpath, educational facility [kindergarten or school], and sports facility), and circumstances surrounding the fracture. Children were assigned to a trauma level category based on a modified Landin's description [15] that considers the height of the fall and the landing surface [16,17], the physical activity engaged in, and whether or not any equipment was being used (Table 2). The occurrence of a previous accidental fracture confirmed by X-ray ascertained by the parents' report was elicited in the past history. The study also included a questionnaire assessment by parents regarding socioeconomic factors (parents' education) and some behavioural issues about the child, such as sports participation in the previous 12 months, weekly hours of sports activity, and daily hours of sedentary behaviours (sum of time spent in television viewing, computer, and video games). The total daily calcium intake was calculated using a food-frequency questionnaire, specifically established for a pediatric population [18]. The optimal daily calcium intake was defined according to the Italian Society for Human Nutrition guidelines [19].

**Table 1:** Criteria for inclusion and exclusion of patients

inclusion criteria	age ≥ 2 years
	resident in the Campania region
exclusion criteria	fracture due to severe trauma
	presence of any specific pathologic process known to affect bone and mineral metabolism
	presence of any specific treatment known to affect bone and mineral metabolism

**Table 2:** Descriptive categories of Landin's modified trauma levels (15)

Slight trauma
Falling to the ground from < 0.5 m (standing height)
Falling to a resilient surface (rubber or sand) from 0.5-3 m
Falling from a bed or cot
Playing injuries, including playground scuffles
Low-energy sporting injuries, such as ball sports, judo, karate, wrestling, and gymnastics
Moderate trauma
Falling to concrete or other non-resilient surface from 0.5-3 m
Falling from a bunkbed
Baby being dropped to the floor by an adult
Falling downstairs
Falling from a bicycle
Falls while moving on skateboards, skis, rollerblades, or skates

Severe trauma
Falling from a height exceeding 3 m
All traffic accidents not already mentioned
Being hit by a moving heavy object

### Statistical analysis

The confidence interval estimation performed to determine the sample size indicated that a size of 127 produced a 99% confidence interval equal to the sample proportion plus or minus 0.05 when the estimated proportion was 0.05 (according to a recent estimated incidence of 5% of fractures in children < 14 years of age [4]). All statistical analyses were carried out using the Statistical Package of Social Sciences (SPSS, Windows release 15.0; Chicago, IL, USA). A p value <0.05 was considered significant.

The results are reported as the mean ± SD. All of the continuous variables had a normal distribution, except for BMI-SDS, time spent in sports activities, and sedentary behaviors. Therefore, an independent sample t-test or a one-way ANOVA with a *post hoc *Bonferroni test were used for parametric variables, while the Mann-Whitney U test or the Kruskall-Wallis test were used for non-parametric variables. A chi-square test was used for categorical variables. Logistic regression analysis was performed to determine relationships between individual and lifestyle variables and fracture recurrence. The outcome variable was fracture recurrence, while the independent variables were gender (coded as 1 for boys and 2 for girls), age, BMI-SDS, adherence to calcium intake recommendations (0 = not adherent; 1 = adherent), time spent in sports activities, or in sedentary behaviours.

## Results

### Prevalence of fractures and lifestyle factors by age group and gender

Three-hundred eighty-two children were enrolled in the study. There were 261 boys (68.3%) and 121 girls (31.7%), with a mean age of 8.8 ± 2.9 years (range, 2-14 years). According to the age groups, there were 76 (19.9%) pre-school children, 199 (52%) school children, and 107 (28.0%) adolescents. The demographic features are shown in Table 3. Males were at a higher risk of fracture than females in every age group, with a prevalence progressively increasing with age (p < 0.001). No difference was found among the three age groups regarding BMI-SDS and parental educational level. The highest frequency of fractures occurred at 12 years of age in boys (15.3%) and 9 years of age in girls (13.2%); the lowest frequency of fractures occurred at 2 years of age in boys (2.7%) and 3 years of age in girls (2.5%). The percentage of subjects fulfilling the daily calcium recommendations significantly decreased with age (pre-school children [86.8%], school children [61.3%], and adolescents [31.8%], p < 0.001), while weekly time spent in sports activities significantly increased (pre-school children [0.38 ± 0.83 hrs/week], school children [1.73 ± 2.3 hrs/week], and adolescents [3.1 ± 3.1 hrs/week], p < 0.001). The time spent in sedentary behaviors was significantly higher in adolescents (5.6 ± 2.9 hrs/day) than pre-school children (4.3 ± 2.9 hrs/day) and school children (4.7 ± 2.8 hrs/day; p < 0.003). No gender difference in any of these behaviors existed in pre-school children, while significant differences existed in both school children in whom males were more sedentary than girls (p = 0.001), and in adolescents in whom males were more physically active (p < 0.02) and more sedentary than girls (p = 0.027; Table 4).

**Table 3:** Demographic features of subjects with fractures in the three age groups

	PRE-SCHOOL CHILDREN	SCHOOL CHILDREN	ADOLESCENTS
N	76	199	107

Boys/girls n (ratio)	42/34 (1.2)	128/71 (1.8)	91/16 (5.7)**

Age (years)	4.38 ± 1.1	8.62 ± 1.4	12.29 ± 0.8

Height (cm)	113.3 ± 11.5	135.8 ± 13.9	152.8 ± 16.9

Weight (kg)	23.5 ± 7.1	38.3 ± 11.28	54.7 ± 11.9

BMI (kg/m^2^)	18.3 ± 4.3	20.2 ± 4.28	22.9 ± 4.1

BMI- SDS	0.93 ± 2.0	1.09 ± 1.10	1.16 ± 0.99

Father's education level n (%)			
Elementary	4 (6.1)	22 (11.7)	6 (6.7)
Middle school	29 (43.9)	80 (42.8)	41 (46.1)
High school	28 (42.4)	69 (36.9)	34 (38.2)
Degree	5 (7.6)	16 (8.6)	8 (9)

Mother's education level n (%)			
Elementary	6 (9.2)	21 (11)	9 (10)
Middle school	24 (36.9)	80 (42.1)	39 (43.3)
High school	30 (46.2)	78 (41.1)	30 (33.3)
Degree	5 (7.7)	11 (5.8)	12 (13.3)

** p < 0.001 pre-school children versus school children and adolescents; children versus adolescents

**Table 4:** Gender comparison of the characteristics of fractures, and nutritional and behavioural habits in the three age groups

	PRE-SCHOOL CHILDREN			SCHOOL CHILDREN			ADOLESCENTS		
	**Males**	**Females**	***p***	**Males**	**Females**	***p***	**Males**	**Females**	***p***

Low energy trauma (%)	66.7	61.3	*0.635*	79.8	61.8	*0.007*	72.6	30.0	*0.006*

Upper limb (%)	91.7	74.2	*0.054*	86.9	86.4	*0.920*	79.5	75.0	*0.723*

Adherence to calcium intake recommendations (%)	83.8	90.6	*0.400*	59.0	65.2	*0.398*	31.8	33.3	*0.904*

Sports participation (hours/week)	0.4 ± 0.9	0.3 ± 0.7	*0.413*	1.6 ± 1.9	2.0 ± 2.9	*0.173*	3.4 ± 3.2	1.7 ± 2.7	*0.043*

Sedentary behaviours (hrs per day)	4.4 ± 3.2	4.2 ± 2.5	*0.735*	5.2 ± 2.9	3.7 ± 2.1	*0.001*	5.9 ± 3.0	4.1 ± 2.1	*0.020*

### Dynamics of fractures

The dynamics of fractures were known in 359 children (93.9%). Fractures due to low-energy trauma occurred in 252 subjects (70.2%). After stratification by gender and age class, low-energy trauma was more frequent in male school children and adolescents (p = 0.007 and p = 0.006, respectively, table 3), while no difference existed in pre-school children.

### Anatomic sites of fractures

The prevalence of fractures according to the anatomic site is shown in table 5. Except for two cases of clavicular fractures, the near totality of injuries involved the upper (84.1% cases) or lower limb (15.9%). The net prevalence of the upper limb over the lower limb was independent of age group. A slight gender discordance existed only in pre-school children in whom upper limb fractures were more frequent in boys than girls (p = 0.054). A further distinction based on upper arm, forearm and wrist, and hand showed significant differences between genders only in pre-school children (p = 0.001) in whom the upper arm was predominantly involved in girls (73.9%) and the forearm and wrist in boys (63.6%).

**Table 5:** Distribution of fractures among the different sites

	number	%
Distal radius and physis	116	30.4

Radial shaft	92	24.1

Elbow area (distal humerus, proximal radius, and ulna)	70	18.3

Humerus (proximal and shaft)	33	8.6

Hand (carpals, metacarpals, and phalanges)	8	2.1

Clavicle	2	0.5

Tibial shaft	22	5.8

Foot (metatarsal and phalanges)	17	4.4

Ankle (distal tibia)	16	4.2

Femur (neck and shaft)	5	1.3

Other	1	0.2

### Locations of fracture occurrences

**Figure 1:**
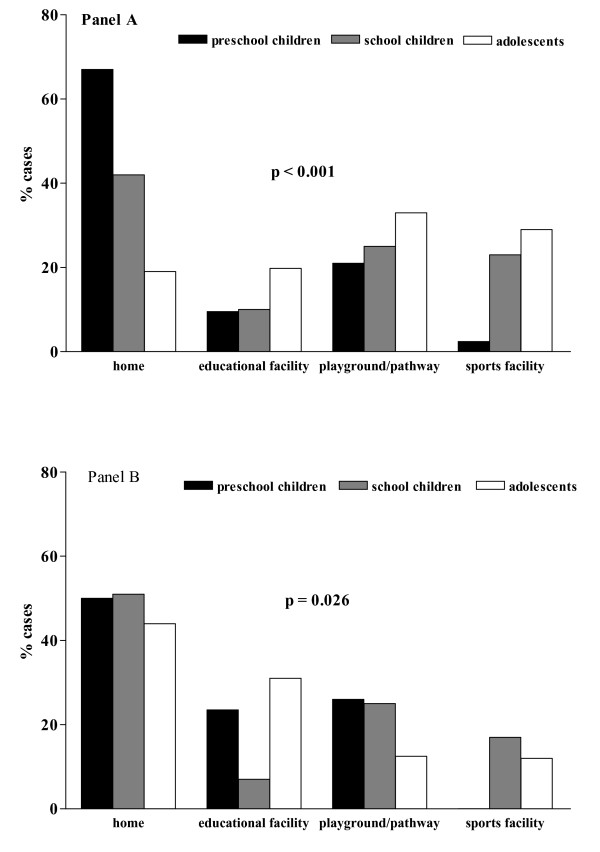
**Main locations of injury in the different age groups (panel A, boys; panel B, girls)**.

The home was the main location (n = 159 [41.6%]), followed by the playground and footpath (n = 100 [26.2%]), sports facility (n = 70 [18.3%]), and educational facility (n = 53 [13.9%]). Location was separately analyzed by gender and age. In males the percentage of fractures occurring in the home significantly decreased with age, while the percentage of fractures occurring in educational facilities, playgrounds and footpaths, or in sports facilities increased (p < 0.001; Figure 1, panel A). In females the home represented the most frequent location at any age, while fractures in the playground and footpath or sports facility significantly decreased with age (p = 0.026; Figure 1, panel B) and a U-shaped curve was observed regarding educational facilities. No difference between genders existed in each age group, except in adolescents, in whom the playground and footpath was the location more frequently reported by males (33.0%) and the home was more frequently reported by females (43.8%, p = 0.048).

### Recurrent fractures

Eighty-eight subjects (23.2%) sustained one or more fractures in the past that were ascribed by parents to accidental injuries. The percentage of recurrent fractures increased from pre-school children to adolescents (from 7.9% to 40.6%, p = 0.001), with a similar trend in both genders. Subjects with recurrent fractures did not differ from those reporting only one fracture with respect to BMI-SDS, time spent in sports activities, or sedentary behaviors (data not shown). Fractures caused by low-energy trauma occurred in 74.7% of subjects with recurrent fractures and 68.7% of subjects reporting only one fracture (p not significant). Fifty-six percent of subjects with recurrent fractures did not fulfill the daily recommendations of calcium intake in comparison with 37% of subjects who reported only one fracture (p = 0.001). In order to exclude confounders, logistic regression analysis was used in which recurrence of fractures was the independent variable and age, gender, BMI-SDS, adherence to daily calcium intake recommendations, weekly time spent in sports activities, and daily time spent in sedentary behaviors were the dependent variables. Only older age was independently associated with recurrent fractures [Exp(B), 1.287; 95% CI, 1.146-1.445, p < 0.001, table 6].

**Table 6:** Variables independently associated with age at fracture occurrence by multiple regression analysis in the entire sample of patients

Independent Variable: Age at fracture occurrence			
***Dependent variables***	**B Coefficients**	**SE**	**p**

Gender (1 = Male, 2 = Female)	-.683	0.29	.018
BMI-SDS	0.208	0.10	.042
Calcium intake (0 = not adherent, 1 = adherent)	-2.218	0.27	.000
Sports activities (hrs/week)	0.357	0.05	0.000
Sedentary behaviors (hrs/day)	0.166	0.05	.001

## Discussion

It has been reported that fractures are a common event in childhood [1-3], with considerable variations in the incidence rate from 1.2% to 5% among different studies [4,20,21]. This variability may depend on the child's condition, age, and social and environmental factors. Few data are available regarding the epidemiology of fractures in the various periods of pediatric ages [21]. These periods are characterized by different stages of physical, cognitive and social development, and may obviously explain the varying pattern of injuries across age groups [21,22]. We enrolled a population of patients receiving treatment for fractures caused by slight or moderate dynamics. In order to describe possible differences among the various developmental stages and define individual and lifestyle determinants of fractures according to age, this population was divided in pre-school children, school children, and adolescents. Our data indicate that males were at higher risk of fractures than females, more than two-thirds of injuries occurred after low-energy trauma, and the upper limb was more frequently involved. With increasing age, the male/female ratio and time spent in sports participation increased, while calcium intake and time spent in sedentary behaviors decreased. A gender discordance was demonstrated in pre-school children with respect to the anatomic location and in school children and adolescents with respect to the dynamics. In the adolescent group, males were not only physically more active than females, but also more sedentary. Gender differences in the incidence of fractures in the pediatric age group are well-known. The overall percentage of children from 0-16 years of age sustaining at least 1 fracture is higher (42%) in boys than girls (27%) [23,24], and the peak incidence is roughly 3 years earlier in girls than boys (11 and 14 years, respectively) [3]. Similarly, our data confirmed that the overall fracture prevalence was higher in boys, independent of age, but the peak frequency occurred 2 years earlier than previous studies reported (9 years in girls and 12 years in boys). The increase in fracture rate during the pubertal years has been explained by a discrepancy between height gain and the accrual of bone mineralization [25]. Since the onset of puberty progressively anticipated in the last century in several European countries, including Italy [26], the 2-year anticipation of the peak frequency of fractures may be explained by the earlier peak height velocity associated with pubertal growth. We found that the male-to-female ratio significantly increased from pre-school children (1.2) to adolescents (5.7) in agreement with previous reports [27,28]. We did not find any difference in the parental educational level among the three age classes. The relationship between socioeconomic status and the risk of fractures has been analyzed with contradictory evidence. While unintentional home injuries in pre-school children is related to the main caregiver's level of education [29], no clear evidence of a socioeconomic gradient in the total incidence of fractures has been shown in childhood [8,15,30,31]. Interestingly, Williams et al. [32] reported that parental socioeconomic status was related to the circumstances in which injury events occurred in adolescents, influencing the extent and type of the risk behaviours. Seasonal variation in the incidence of pediatric fractures has been reported in several studies, with the highest peak found in the warmer months [21,33]. Subjects in our investigation were enrolled in the first 6 months of the year. A full-year review would have been valuable in order to exclude a possible enrollment bias; however, the weather in our region does not substantially influence behaviours during leisure time. Moreover, the admissions for fractures were equally distributed in the first and second semesters of the year according to the hospital report.

We showed that in 77% of cases fractures were ascribed to low-energy trauma (mainly falls) that occurred more frequently in males in school children and adolescents. Similarly, Rennie et al. [21], who analyzed the basic epidemiology for different mechanisms of fracture in British children, reported that falls accounted for 57% of all fractures, occurred at a younger age and prevailed in males.

Increased participation in both organized and informal sports, as well as the overall high levels of physical activity during adolescence, has been previously advocated to explain the increased incidence of fractures in adolescents [21,34-36]. In particular, gender difference in the incidence of injuries may be explained by age-related changes in behaviors, such as participation in activities with increased physical risks by males [37]. In a population-based case control study, Ma and Jones [6] reported that participation in sports increased the risk of upper limb fractures in boys and decreased the risk of upper limb fractures in girls, even for the same sport. This effect was independent of bone mass, suggesting gender heterogeneity in the approach to sports, and implies that the beneficial effect of physical activity on bone health [38,39] can be hindered in males, who probably are engaged in physical activities with higher potential for trauma. Indeed the relationship between physical activity, bone mass, and childhood fracture risk is complex. A positive association between vigorous physical activity and childhood fractures that was independent of bone mass was demonstrated by Clark et al. [15]. Participation in vigorous physical activity or contact sport may increase bone mass, but does not protect from the risk caused by increased exposure to injuries. The influence of physical activity on fracture risk is determined by its net effect on fall-related trauma and bone strength. Therefore, as motor ability increases, the involvement in physical activities increases and the risk of injuries increases, particularly in boys [40]. In agreement with these previous reports, we found that adolescents sustaining a fracture spent higher amounts of time in organized physical activity than their counterparts in pre-school children or school children; in addition, males were more physically active and sedentary than females. A previous study reported a dose response association between time spent in television, computer, and video viewing and wrist and forearm fracture risk in both genders [6]. It would be expected that sedentary behaviours would reduce levels of physical activity and consequently lower the risk of trauma. Indeed, several studies have not demonstrated any negative relationship between video exposition and moderate or vigorous leisure-time physical activity, indicating that these behaviours may co-exist [41]. The increased time spent watching television or playing computer games may lead to unhealthy behaviours, such as increased consumption of energy**-**dense snack foods or carbonated beverages, obesity, or aggressive behaviour, conditions that may place children at greater risk of fractures [42,43].

Regarding the anatomic site, the upper limb is more frequently involved in pediatric fractures, accounting for approximately two-thirds of all fractures [20]. We confirmed that the upper limb was more frequently involved at any age, representing indeed > 80% of fractures, in agreement with a previous epidemiologic study performed in various age groups [21]. This higher prevalence may be explained by the fact that 77% children in our study reported low-energy fractures, the main cause being represented by falls. Indeed, the arm is more frequently involved after a fall in children > 5 years of age [44]. In pre-school children, the upper limb was involved in 92% of cases in boys and 74% of cases in girls, despite no difference in dynamics between genders. This finding is in agreement with a larger survey on the incidence pattern for different fracture sites by age and gender in which sexual dimorphism existed for fractures of the tibia/fibula [3]; in fact, a higher incidence of fractures at this site existed in girls < 6 years of age and in boys > 14 years of age. Age-related differences at the upper site have been also described; specifically, humeral fractures tend to peak first (6-7 years in both genders) followed by radius-ulna fractures (10-11 years in girls and 12-13 years in boys), while carpal fractures peak later (12-13 years in girls and 14-15 years in boys). We found that the frequency of upper arm, forearm and wrist, and hand fractures differed between genders only in pre-school age children, when females predominantly sustained upper arm fractures (73.9%) and boys sustained forearm and wrist fractures (63.6%). These discrepancies can probably be explained by different specific causes related to fall characteristics.

Regarding the locations where fractures commonly occur, it has been reported that the home accounts for 37% of all fractures in children, while the school represents 20% [23]. In our experience as well, the home was the place where fractures most frequently occurred, and locations changed among age groups and gender. These findings are not surprising and reflect the amount of time that pre-school children spend at home in comparison with older children and adolescents who gradually spend a greater amount of their active time outside the home [22]. Significant differences between genders were found only in adolescents (p = 0.048) because boys mainly sustained fractures in the playgrounds and footpaths and girls sustained fractures at home.

It is well-known that children who experienced one fracture tend to be at increased risk of repeated fractures [12,13] and have lower bone mineral density and accretion than their peers [9,45,46]. We found that 23.2% of otherwise healthy children sustained one or more previous fractures, with an increasing percentage from pre-schoolers to adolescents (from 7.9% to 40.6%, p = 0.001) and no difference between genders. This fact is not surprising, since with increasing age there is a longer exposure time for injuries. We excluded underlying pathology or chronic illnesses that may have predisposed the subjects to reduced bone mineralization, although we could not determine whether or not these children were "accident prone," or lived in a dangerous environment. No one was suspected to be victim of physical abuse. Insufficient calcium intake was found in a large number of children with recurrent fractures. Indeed, low calcium intake has been linked with decreased bone density and fracture risk in children. Children who sustained repeated fractures had total body and lumbar spine bone size and mass significantly lower than controls; they also had a significantly lower intake of milk, lower levels of physical activity, a higher BMI, and a higher consumption of carbonated beverages [9]. We found that the association with low calcium intake disappeared in logistic linear regression analysis, where only age was independently associated with fracture recurrence. Therefore, in our sample, the association of low calcium intake with recurrent fractures was primarily mediated by age.

## Conclusions

The differences which existed in the prevalence of injuries, characteristics, and circumstances across the three age groups may be explained by age- and gender-related changes in behaviors, together with attending different places. Individual and lifestyle factors ascribed to either higher sports activities or sedentary behaviors can in part explain the variability in the occurrence of fractures in older age. The incidence of paediatric fractures can be reduced with public education, implementation of safety strategies, and government legislation. Health care professionals and paediatricians can be instrumental in reducing the incidence of paediatric injuries by participating in child education, research, and programs that promote safe play.

## Competing interests

The authors declare that they have no competing interests.

## Authors' contributions

GV, PG, and GL provided substantial contributions to the conception and design of the study, definition of the objectives, development of the questionnaire, and analysis and interpretation of data; they revised the paper critically for important intellectual content and gave their final approval of the version to be published.

GV, FG, and VDO provided substantial contributions to background analysis and literature research, analysis, and interpretation of data, drafting the manuscript, and gave their final approval of the version to be published.

CM and MC provided substantial contributions to acquisition of data, parent interviews, and development of the database; they inputted the data in dedicated software, contributed to drafting the manuscript, and gave their final approval of the version to be published.

## Pre-publication history

The pre-publication history for this paper can be accessed here:

http://www.biomedcentral.com/1471-2458/10/656/prepub
